# Combined Post-traumatic Pseudoaneurysms of the Superior Gluteal and Aberrant Obturator Arteries: A Case Report

**DOI:** 10.7759/cureus.115456

**Published:** 2026-08-30

**Authors:** Lahcen Kaddouri, Youssef Benhmammouche, Kenza Benzmane, Youssef Belchiti, Abdelkarim Kharroubi

**Affiliations:** 1 Department of Vascular and Endovascular Surgery, University Hospital Center (CHU) Souss Massa, Agadir, MAR; 2 Department of Vascular and Endovascular Surgery, Medicine and Pharmacy, Université Ibn Zohr, Agadir, MAR

**Keywords:** aberrant obturator artery, anatomical variation, hemorrhage, pseudoaneurysm, superior gluteal artery

## Abstract

Post-traumatic pelvic vascular injuries are rare complications of blunt trauma, but they can be serious. Arterial pseudoaneurysms are rare and may be difficult to diagnose because of delayed presentation and the complex pelvic vascular anatomy. Post-traumatic pseudoaneurysms of the superior gluteal artery, as those associated with particular anatomical variants like aberrant obturator arteries, are rare. They can further complicate diagnosis and management. We report the case of a 14-year-old patient admitted with abdominal pain and signs of sepsis after pelvic trauma caused by a fall. An angiogram revealed the presence of bilateral retroperitoneal hematomas without active bleeding. Due to a deterioration in the patient’s clinical condition, an exploratory laparotomy was performed to drain the hematomas and irrigate the peritoneal cavity; no active bleeding was observed. Postoperative CT angiography (CTA) demonstrated two pseudoaneurysms arising from the superior gluteal artery and the unilateral aberrant obturator artery. The patient remained hemodynamically stable, and a conservative management strategy accompanied by close clinical and radiological monitoring was established, leading to spontaneous thrombosis of the two pseudoaneurysms and a favorable clinical outcome.

## Introduction

The obturator artery typically arises from the anterior division of the internal iliac artery. It courses along the lateral pelvic wall in close relationship with the obturator nerve and vein before passing through the obturator canal. Within the pelvis, it gives rise to iliac, vesical, and pubic branches. Distal to the obturator canal, it divides into anterior and posterior branches that supply the muscles of the medial compartment of the thigh [1]. The so-called “aberrant” obturator artery has a variable origin and may arise from either the external iliac artery or the inferior epigastric artery. The obturator artery most commonly arises from the anterior division of the internal iliac artery (59.3%), whilst an aberrant origin from the inferior epigastric artery is observed in nearly a quarter of cases (23.4%). It is of major clinical importance due to the high risk of injury, with high mortality due to hemorrhage and the difficulty of achieving hemostasis during pelvic trauma or surgical procedures, particularly during inguinal hernia repairs, orthopedic procedures, and pelvic trauma [2].

The superior gluteal artery is a terminal branch of the posterior division of the internal iliac artery. The main arteries to the gluteal region are the inferior and superior gluteal arteries. It was once reported that the superior gluteal artery supplied blood to the superior half of the gluteus maximus and the inferior gluteal artery supplied blood to the inferior half of the muscle. Pseudoaneurysms involving these arteries are rare and are most commonly post-traumatic [3]. Angiography and embolization have achieved remarkable advances in the evaluation and initial management of arterial injuries, leading to improved survival rates in hemodynamically unstable patients [4].

Multidetector computed tomography angiography (CTA) is currently considered the imaging modality of choice for the evaluation of pelvic vascular injuries. It enables rapid and accurate detection of pseudoaneurysms, active contrast extravasation, vascular anatomical variants, and associated hematomas. Furthermore, multiplanar and three-dimensional reconstructions enhance visualization of the vascular anatomy and facilitate therapeutic planning [5]. Although post-traumatic pseudoaneurysms of the pelvic arteries are rare, their occurrence in the setting of uncommon arterial anatomical variants may pose additional diagnostic and therapeutic challenges.

We report the case of a patient who presented with two pseudoaneurysms: one involving a rare arterial anatomical variation of the right aberrant obturator artery originating directly from the right external iliac artery, characterized by bilateral symmetry of this variant, and associated with a pseudoaneurysm of the superior gluteal artery. To our knowledge, this vascular association has been reported only exceptionally in the literature. The uniqueness of this case also lies in its favorable outcome following conservative management, with spontaneous thrombosis of both pseudoaneurysms, highlighting the pivotal role of CTA in the diagnosis, follow-up, and therapeutic decision-making of these rare vascular injuries.

## Case presentation

A 14-year-old patient with no significant previous medical history reported abdominal pain radiating to the pelvis that began seven days prior to admission following a fall from height with direct impact to the pelvic region.

At admission, the patient presented with pallor, tachycardia at 100 beats/min, hypotension (100/70 mmHg), and a fever of 39°C consistent with septic shock. Abdominal examination revealed generalized guarding, more pronounced in the right flank. Initial laboratory investigations demonstrated severe anemia, with a hemoglobin level of 7.5 g/dL, marked leukocytosis (59.28 × 10⁹/L) predominantly due to neutrophilia (51.90 × 10⁹/L), thrombocytosis (642 × 10⁹/L), blood urea of 0.95 g/L, and a serum creatinine level of 11 mg/L (Table 1).

**Table 1:** Baseline vital signs and laboratory findings at admission

Parameter	Value
Heart rate	100 beats/min
Blood pressure	100/70 mmHg
Body temperature	39°C
Hemoglobin	7.5 g/dL
White blood cell count	59.28 × 10⁹/L
Neutrophil count	51.90 × 10⁹/L
Platelet count	642 × 10⁹/L
Blood urea	0.95 g/L
Serum creatinine	11 mg/L

Initial contrast-enhanced abdominopelvic CT was performed using helical acquisition during the portal venous phase. It demonstrated multiple collections of hematoma density containing a few internal air bubbles and showing mild peripheral enhancement after contrast administration. The largest collection involved the right lumbar psoas muscle, measuring approximately 180 × 73 × 53 mm, extending posteriorly into the retroperitoneal space and exerting a mass effect on the rectosigmoid junction and urinary bladder, with associated right ureteropyelocaliceal dilatation. A second collection involved the right iliopsoas muscle, measuring 123 × 74 × 25 mm and extending inferiorly to the insertion of the lesser trochanter. Additional collections were identified within the right piriformis muscle (63 × 40 × 37 mm) and in the right subcolic region (59 × 41 × 46 mm). A right colonic wall perforation was initially suspected. A small-volume retroperitoneal and perihepatic (Morrison's pouch) fluid collection was also present. No active contrast extravasation was identified on the portal venous phase images. Overall, the imaging findings were considered consistent with multiple infected hematomas involving the right psoas muscle and the right subcolic region, associated with a small-volume intra- and retroperitoneal fluid collection (Figure 1).

**Figure 1:**
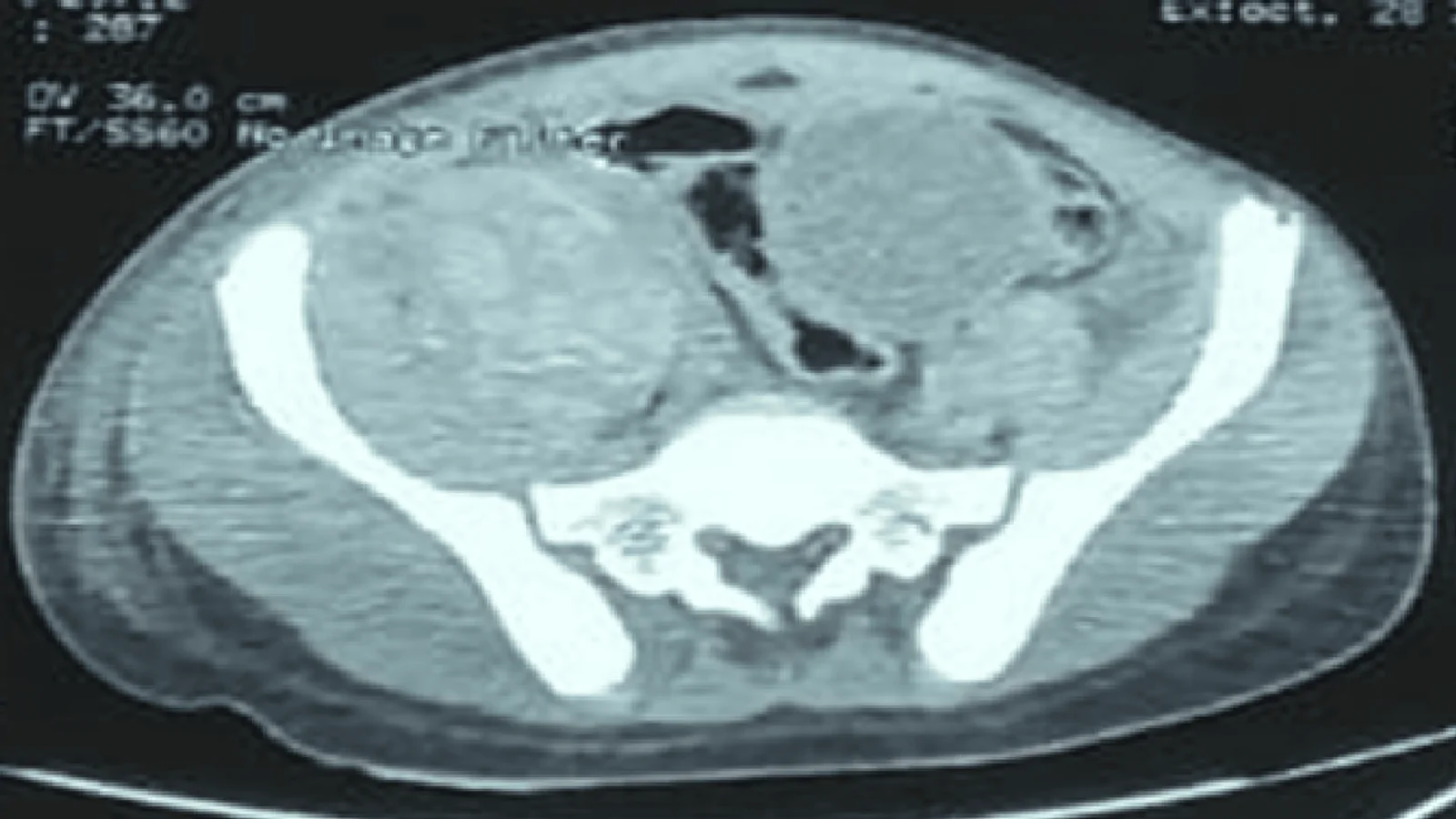
Axial contrast-enhanced abdominopelvic CT image acquired during the portal venous phase Demonstrating a large right psoas hematoma extending into the retroperitoneal space, with mass effect on adjacent pelvic structures. No active contrast extravasation was identified.

The patient underwent exploratory laparotomy, which revealed a large right retroperitoneal hematoma associated with a left retroperitoneal hematoma, both non-expanding. Careful evacuation of the accessible hematoma was performed, followed by extensive lavage with normal saline until clear fluid was obtained, without evidence of active bleeding or purulent collection.

During the postoperative follow-up, abdominal pain and guarding persisted. The patient remained conscious (Glasgow Coma Scale score 15/15) and hemodynamically and respiratory stable: heart rate 90 beats/min, normal blood pressure (13/7), respiratory rate 17 cycles/min, and afebrile (36.5°C). There were no neurological deficits, no signs of peritoneal irritation, and no active external bleeding.

On day 2 after admission, CTA was performed and revealed two pseudoaneurysms: a pseudoaneurysm of the right aberrant obturator artery directly from the right external iliac artery supplying a pelvic hematoma located in the right iliac fossa, in a subperitoneal position displacing the bladder superiorly and to the left (Figure 2), and a pseudoaneurysm of the right superior gluteal artery feeding a presacral hematoma with anterior displacement of the rectum (Figures 3, 4).

**Figure 2:**
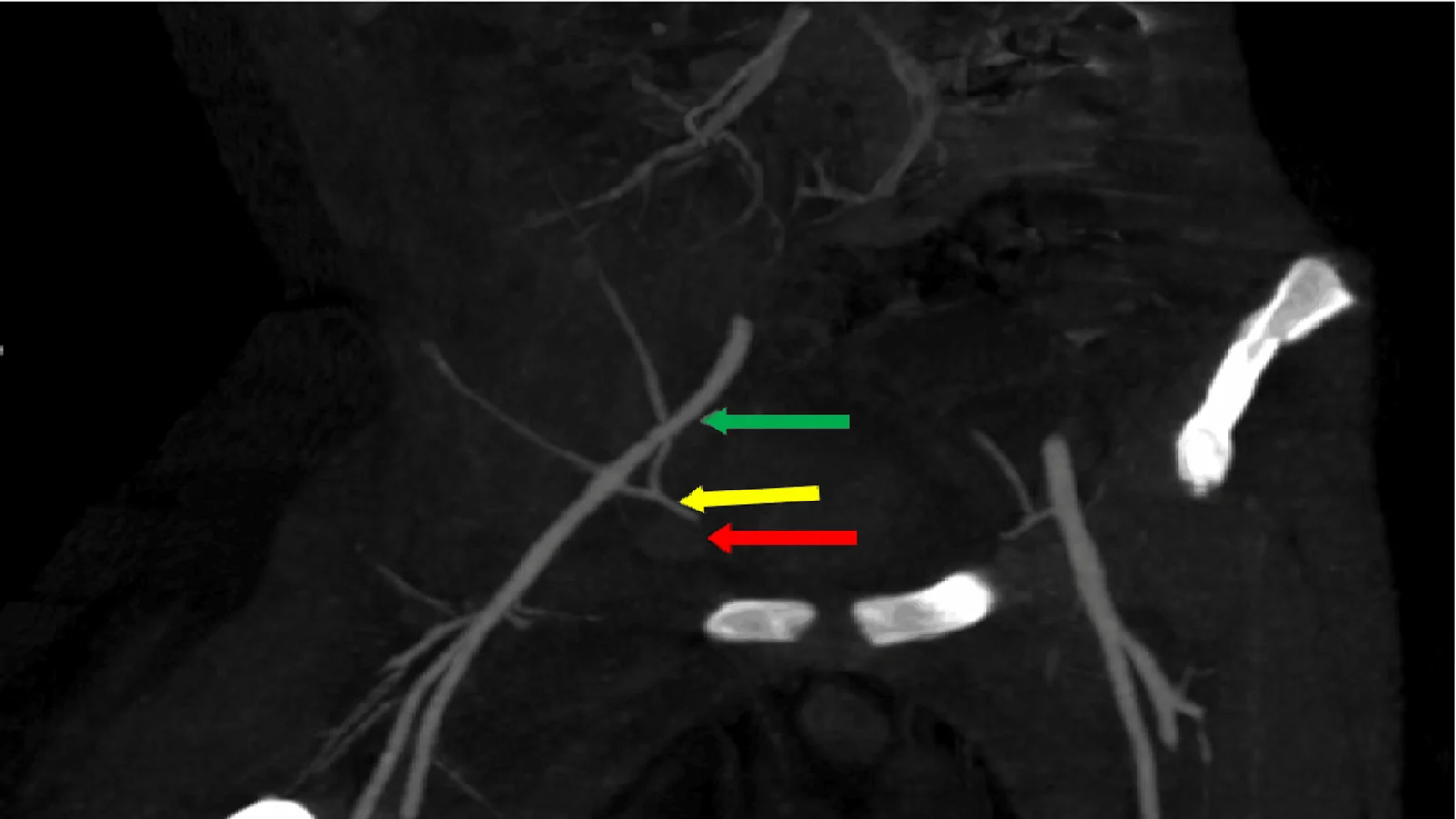
Coronal multiplanar reconstruction (MPR) of contrast-enhanced CT angiography acquired during the arterial phase Demonstrating a post-traumatic pseudoaneurysm (red arrow) arising from the right aberrant obturator artery (yellow arrow), originating from the right external iliac artery (green arrow). The reconstruction clearly demonstrates the abnormal arterial origin and the relationship between the pseudoaneurysm and the external iliac arterial system.

**Figure 3:**
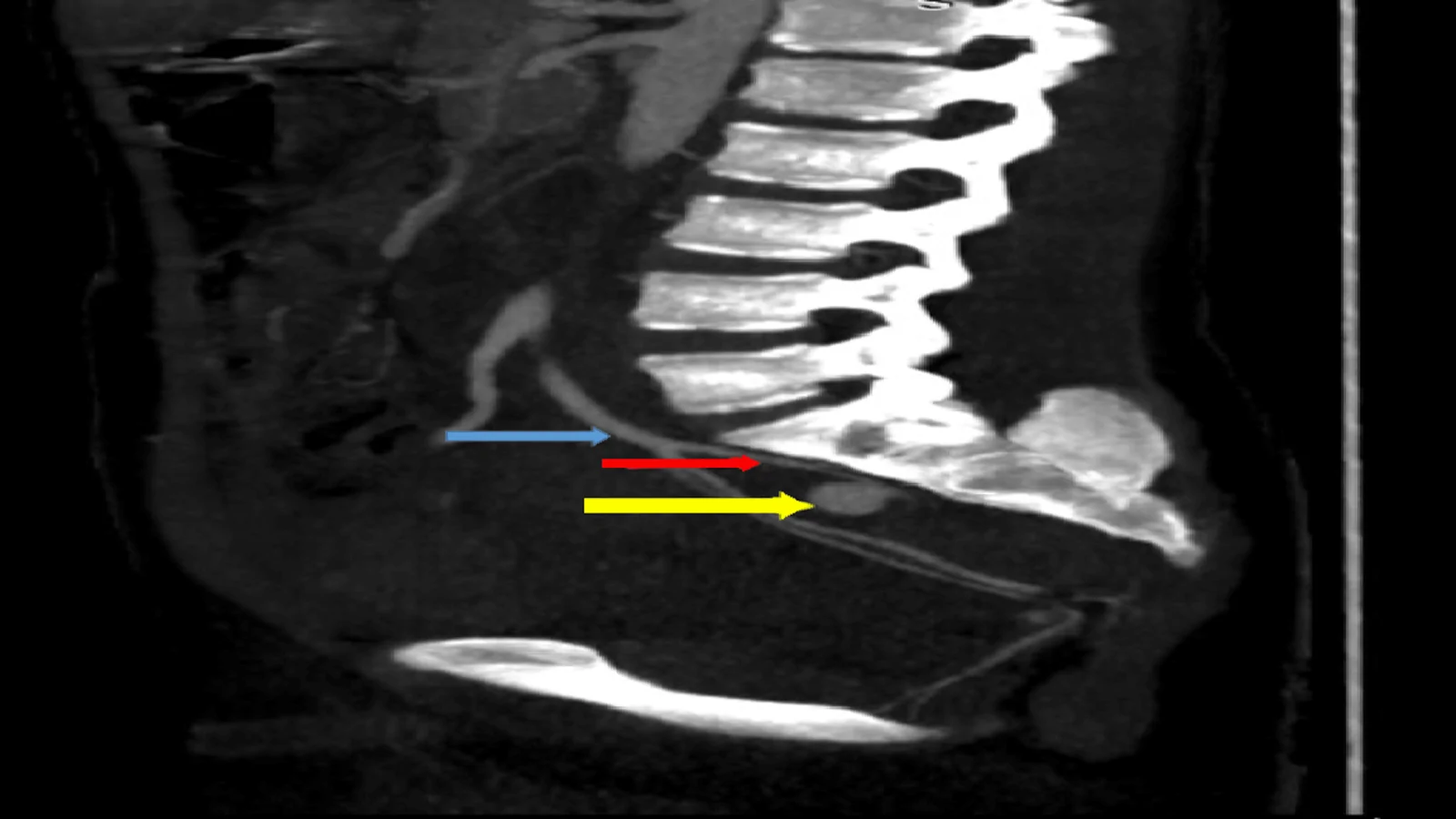
Sagittal multiplanar reconstruction of contrast-enhanced CT angiography acquired during the arterial phase Demonstrating a post-traumatic pseudoaneurysm (yellow arrow) arising from the right superior gluteal artery (red arrow), a branch of the internal iliac artery (blue arrow).

**Figure 4:**
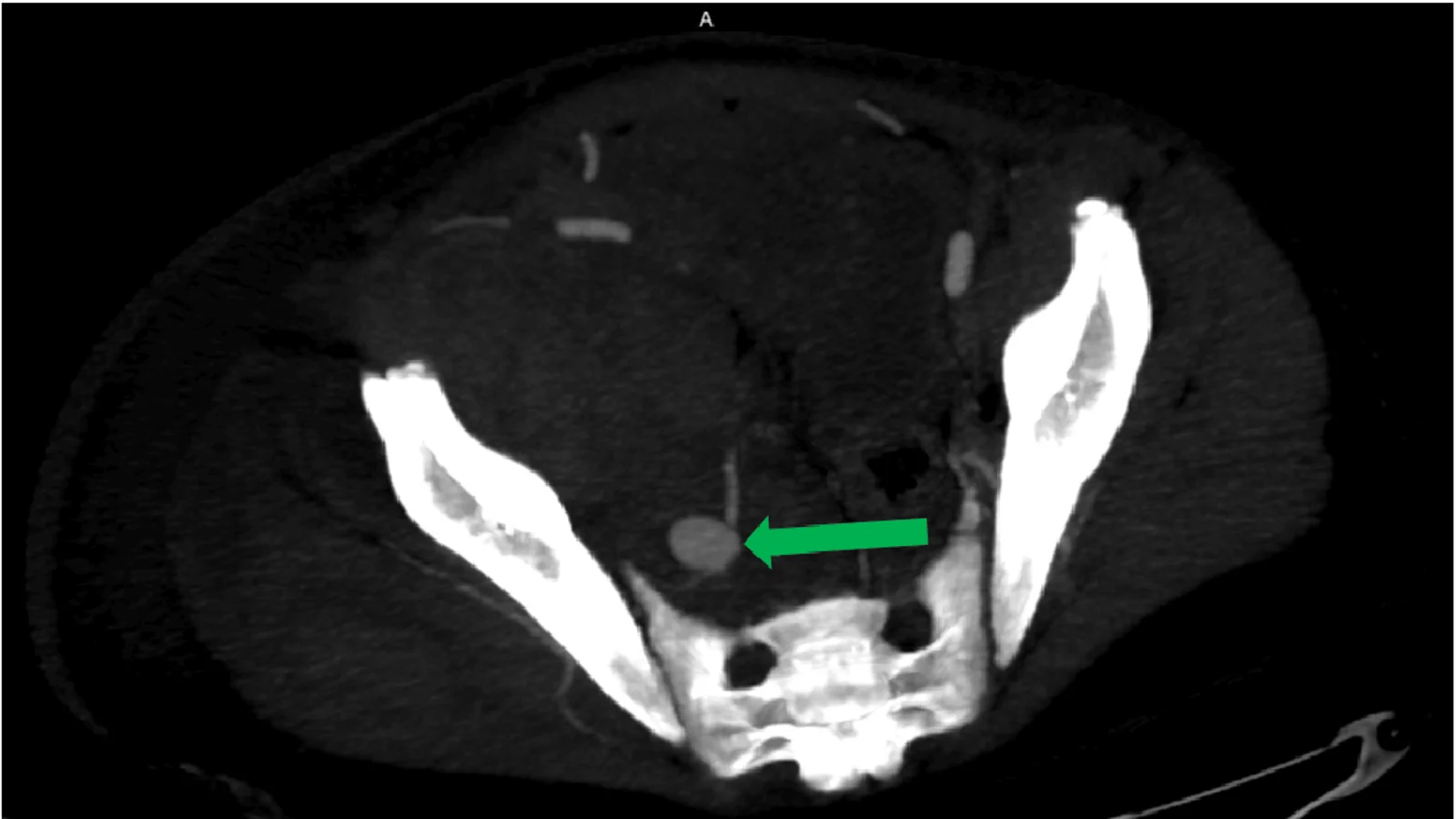
Coronal multiplanar reconstruction of contrast-enhanced CT angiography acquired during the arterial phase Demonstrating a post-traumatic pseudoaneurysm of the right superior gluteal artery (green arrow).

The differential diagnosis of post-traumatic pelvic vascular lesions includes active arterial hemorrhage, arteriovenous fistula, vascular malformations, and organized hematoma. CTA demonstrated the absence of active contrast extravasation, excluding ongoing arterial bleeding, while the lack of early venous opacification argued against an arteriovenous fistula. Furthermore, the delayed post-traumatic presentation and characteristic imaging features were not compatible with a congenital vascular malformation or an isolated organized hematoma.

Clinical evolution was marked by resolution of pain and improvement in general condition. On day 7 after admission, follow-up CTA demonstrated spontaneous thrombosis of both pseudoaneurysms, with cessation of bleeding from the right superior gluteal and aberrant obturator arteries, along with persistence of stable retroperitoneal and psoas hematomas extending toward the root of the thigh.

Therapeutic decision

The decision to opt for conservative management was based on several factors. Firstly, the patient was clinically stable, with no signs of active bleeding or hemodynamic instability, which made close monitoring the preferred option. Secondly, the initial radiological findings suggesting spontaneous thrombosis of the lesions reduced the need for immediate invasive intervention.

The decision on treatment also took into account logistical and clinical factors, including the limited availability of interventional radiology facilities in an emergency, the patient’s stable general condition, which did not warrant invasive treatment as a first-line option, and the need to obtain the patient’s informed consent following a discussion of the available treatment options. Overall, these considerations led to the decision to adopt a conservative strategy combined with rigorous clinical and radiological monitoring.

Outcome

During follow-up consultations, the patient remained clinically stable, with no fever, no abdominal or lower back pain, and no signs of complications. Repeated laboratory investigations showed no inflammatory syndrome or infectious abnormalities.

Contrast-enhanced thoraco-abdominal CT performed one month after discharge demonstrated persistent right retroperitoneal collections. The largest collection, located in the posterior pararenal and retroperitoneal spaces, measured approximately 170 × 51 × 100 mm (craniocaudal × transverse × anteroposterior dimensions). A second collection in close contact with the right iliopsoas muscle measured approximately 187 × 45 × 96 mm and extended inferiorly toward the paravesical region. An additional collection involving the right gluteal muscles, located between the gluteus maximus and gluteus medius muscles, measured approximately 11 × 67 mm. No radiological signs of superinfection, such as gas formation, increase in size, or adjacent tissue infiltration, were observed. Overall clinico-biological and radiological findings were consistent with organized, uncomplicated hematomas (Figure 5). Chronological timeline of clinical events, imaging studies, and management is summarized in Table 2.

**Figure 5:**
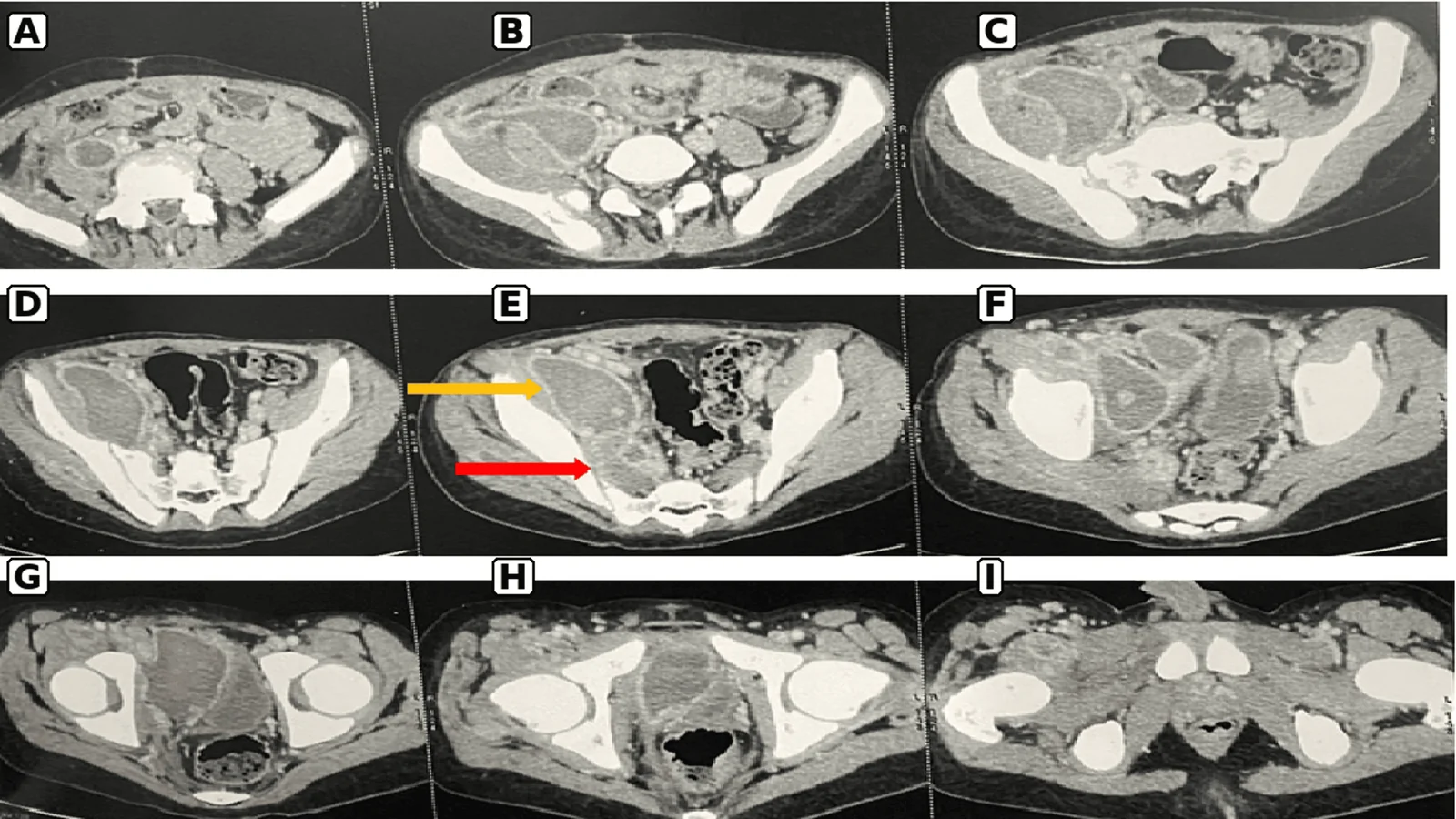
Axial contrast-enhanced CT image A-I: Sequential axial images showing a persistent right iliac fossa/pelvic hematoma (orange arrow) and presacral hematoma (red arrow), without significant interval change in size. The examination was performed using thin-slice helical acquisition following intravenous administration of iodinated contrast material in the arterial phase. Multiplanar reconstructions were additionally obtained to assess the extent of the hematomas.

**Table 2:** Chronological timeline of clinical events, imaging studies, and management

Time point	Clinical events and findings	Investigations/Imaging	Management/Outcome
Day −7	Fall from height with direct pelvic impact, followed by abdominal pain radiating to the pelvis	—	—
Day 0 – Admission	Pallor, tachycardia (100 beats/min), hypotension (100/70 mmHg), fever (39°C), and generalized abdominal guarding, more pronounced in the right flank	Initial laboratory investigations showed severe anemia and marked leukocytosis with neutrophilia and thrombocytosis (Table 1)	Initial clinical management and evaluation
Day 0 – Initial CT	Persistent abdominal pain and clinical signs of infection	Contrast-enhanced abdominopelvic CT in the portal venous phase showed multiple retroperitoneal and muscular hematomas, without active contrast extravasation	Findings initially interpreted as multiple infected hematomas
Day 0 – Exploratory laparotomy	Large right and left non-expanding retroperitoneal hematomas	Intraoperative exploration revealed no active bleeding	Accessible hematoma evacuation and extensive saline lavage
Postoperative period	Persistent abdominal pain and guarding; patient remained conscious and hemodynamically stable, with no neurological deficit, peritoneal signs, or active external bleeding	—	Continued clinical monitoring
Day 2 – Diagnostic CTA	Persistent symptoms despite initial management	CT angiography demonstrated two post-traumatic pseudoaneurysms: one arising from the right aberrant obturator artery and one from the right superior gluteal artery	Conservative management selected based on clinical stability, absence of active bleeding, and available therapeutic resources
Day 7 – Follow-up CTA	Progressive resolution of pain and improvement in general condition	Follow-up CT angiography demonstrated spontaneous thrombosis of both pseudoaneurysms	Continued clinical and radiological surveillance
1 month after discharge	Patient remained clinically stable, afebrile, without abdominal or lower back pain	Contrast-enhanced thoraco-abdominal CT showed persistent but unchanged retroperitoneal collections, without gas, enlargement, or adjacent tissue infiltration	Findings consistent with organized, uncomplicated hematomas

## Discussion

Pseudoaneurysm of the superior gluteal artery

A pseudoaneurysm is a breach involving the entire arterial wall (intima, media, and adventitia), creating a chronic blood collection contained by surrounding tissues. Superior gluteal artery aneurysms, true or false, are rare, accounting for less than 1% of all aneurysms [3]. They are often caused by blunt or penetrating trauma, though they can also follow pelvic surgery, intramuscular injections, or bone marrow biopsy, and more rarely be of infectious, inflammatory, or atherosclerotic origin.

Diagnosis is often delayed because of non-specific symptoms, including neurologic signs (sciatic pain, dysesthesia, muscle atrophy) or vascular signs (pulsatile mass, swelling, bruit) [3,6-8]. In our case, symptoms included abdominal pain radiating to the pelvis.

Diagnostic imaging includes ultrasound, CT, MRI, and angiography [9]. Pelvic CT and MRI also guide treatment strategies. In this case, the pseudoaneurysm was suspected by CT and confirmed by aorto-iliac angio-CT.

Pseudoaneurysms are vascular emergencies due to the risk of rupture and massive hemorrhage [6,7]. They may enlarge, compress adjacent structures, or spontaneously thrombose. In this patient, spontaneous thrombosis and hematoma resorption occurred.

Pseudoaneurysm of an aberrant obturator artery

The obturator artery most frequently originates from the anterior branch of the internal iliac artery. However, anatomical variations, especially origins from the external iliac or inferior epigastric arteries, have been widely reported. Mahato et al. reported an 8% incidence from the inferior epigastric artery; Lau and Lee found 14.81%; Pick et al. reported rates of 22-27.34% [10-13]. Bilateral symmetry aberrant obturator artery was found in only one case in a study of 64 cadavers [2]. 

During the fourth week of embryonic development, the paired dorsal aortae fuse caudally to the tenth dorsal intersegmental arteries, giving rise to the descending aorta. At this stage, each side also contains a paired ventral segmental artery associated with the connecting stalk. During the fifth week, the proximal portion of these arteries establishes an anastomosis with the fifth lumbar intersegmental artery, resulting in the formation of a new arterial stem. The newly formed dorsal root subsequently becomes the dominant arterial pathway, whereas the original ventral root undergoes regression. The dorsal root then contributes to the development of the abdominal and pelvic arterial plexuses. The pelvic plexus subsequently gives rise to the common iliac artery and its major branches, including the external and internal iliac arteries [14-16].

It appears that the obturator artery forms as a result of uneven growth of an anastomosis of the external iliac artery and internal iliac artery. In addition, the obturator artery arises comparatively late to supply the medial side of the thigh. Both the uneven anastomosis growth and its late appearance may explain the occurrence of obturator artery origin and trajectory variations [14-19]. In relation to the uneven anastomosis growth theory, some obturator artery variations, such as its origin from the internal iliac artery posterior division, can be explained as vascular channels for anastomosis that persist in the posterior internal iliac artery division, and those predestined for the obturator artery may have disappeared or degenerated in the anterior division [17-19]. The aberrant obturator artery originating from the deep inferior epigastric artery (DIEA) may be due to the underdevelopment or obliteration of a normal obturator at its origin and an enlargement of an anastomosis between the pubic branches of DIEA and the obturator artery behind the pubic body [20,21]. A dual origin of the obturator artery may be interpreted as the presence of two source channels for the blood flow, one from the internal iliac artery and the other from the DIEA [17,22]. Like all pelvic arteries, the aberrant obturator artery is at risk of injury, with bleeding that can be difficult to identify and treat effectively [8]. In contrast, our patient presented with concomitant pseudoaneurysms in two distinct pelvic arterial territories associated with bilateral symmetrical aberrant obturator arteries.

Previous reports have predominantly described isolated pseudoaneurysms of the superior gluteal artery following pelvic trauma. Dongola et al. reported a post-traumatic superior gluteal artery pseudoaneurysm after minor blunt trauma that was successfully treated by transcatheter embolization [23]. Similarly, Taif et al. described a superior gluteal artery pseudoaneurysm associated with pelvic fracture, which was successfully managed by coil embolization [24]. Babu et al. reported a superior gluteal artery pseudoaneurysm following blunt trauma without associated bony injury; CTA demonstrated the vascular lesion within an expanding gluteal hematoma, and the patient underwent successful angioembolization [25]. Trpeski et al. also reported a delayed presentation of a superior gluteal artery pseudoaneurysm two months after blunt pelvic trauma, with successful treatment by angioembolization [26].

Pseudoaneurysms involving the obturator artery are also uncommon and have been described predominantly in iatrogenic or traumatic settings. A study reported a postsurgical obturator artery pseudoaneurysm successfully treated by superselective transcatheter embolization, emphasizing the importance of recognizing the variable anatomy of the obturator artery and its potential anastomoses with the inferior epigastric artery [27]. Because of these anatomical communications, angiographic evaluation of both the internal and external iliac arterial territories may be necessary when an injury involving the obturator artery or the so-called corona mortis is suspected.

The management of traumatic lesions involving an aberrant obturator artery has similarly favored endovascular embolization. Daeubler et al. described hemorrhage secondary to pelvic fracture caused by an aberrant obturator artery and successfully treated the lesion by coil embolization [28]. More recently, Kim and Lim reported a series of 14 patients with pelvic trauma who underwent transcatheter embolization of the corona mortis/aberrant obturator artery; 12 patients had CT findings of active bleeding, including pseudoaneurysm or contrast extravasation, and technical success was achieved in all patients without procedure-related complications [29]. These findings support selective or superselective embolization as an effective and minimally invasive treatment for clinically significant bleeding arising from an aberrant obturator artery.

Our case differs from these previously reported cases by the simultaneous occurrence of two post-traumatic pseudoaneurysms involving two distinct pelvic arterial territories: the superior gluteal artery and a right aberrant obturator artery. Moreover, the aberrant obturator artery was part of a bilateral and symmetrical anatomical variant.

Conservative management was primarily dictated by the absence of an available endovascular interventional platform at our institution at the time of management. However, the patient remained hemodynamically stable, with no clinical or radiological evidence of ongoing active bleeding. Close clinical and radiological surveillance was therefore undertaken. Follow-up imaging demonstrated progressive and complete spontaneous thrombosis of both pseudoaneurysms, without subsequent hemorrhagic complications.

Although endovascular embolization remains the preferred treatment for most traumatic pelvic pseudoaneurysms, particularly in the presence of active bleeding or hemodynamic instability, our case illustrates that spontaneous thrombosis may occasionally occur in clinically stable patients. Nevertheless, this favorable outcome should not be interpreted as evidence supporting conservative management as a general alternative to endovascular treatment. Rather, it represents an unusual evolution observed under specific clinical and technical circumstances.

An additional distinctive feature was the spontaneous thrombosis of both pseudoaneurysms during conservative management, without subsequent hemorrhagic complications. To our knowledge, the combination of concomitant superior gluteal and aberrant obturator artery pseudoaneurysms following blunt pelvic trauma, associated with spontaneous thrombosis of both lesions, has not been previously reported.

Moreover, both pseudoaneurysms underwent spontaneous thrombosis during conservative management, an outcome that appears less frequently documented in the available literature. This combination of vascular injury, unusual pelvic arterial anatomy, and spontaneous thrombosis represents the main distinctive feature of our case.

Failure to recognize these vascular anomalies can result in inadequate treatment. Surgeons and radiologists must be aware of such variants when managing pelvic trauma.

They are typically secondary to trauma, surgery, or medical procedures involving the pelvic region. Their evolution may lead to thrombosis and spontaneous resolution, rupture, or distal tissue damage from microemboli. Surgeons must remain vigilant about their location and anatomical variants to minimize mortality risk.

## Conclusions

Although rare, post-traumatic pseudoaneurysms of the superior gluteal and aberrant obturator arteries are potentially life-threatening vascular injuries that require a high index of clinical suspicion. This case highlights the value of delayed CTA in establishing the diagnosis when clinical suspicion persists despite initially inconclusive imaging, particularly in the presence of unusual pelvic arterial anatomy. In this hemodynamically stable patient, both pseudoaneurysms underwent spontaneous thrombosis during close clinical and radiological surveillance in a setting where emergency endovascular embolization was not available. This favorable outcome should not be generalized to all traumatic pelvic pseudoaneurysms, for which endovascular embolization remains the preferred treatment when indicated and available. Management should be individualized according to hemodynamic status, imaging findings, risk of ongoing or recurrent hemorrhage, and available therapeutic resources.
